# Effects of Mating and Social Exposure on Cell Proliferation in the Adult Male Prairie Vole (*Microtus ochrogaster*)

**DOI:** 10.1155/2020/8869669

**Published:** 2020-09-22

**Authors:** A. E. Castro, L. J. Young, F. J. Camacho, R. G. Paredes, N. F. Diaz, W. Portillo

**Affiliations:** ^1^Instituto de Neurobiología, Universidad Nacional Autónoma de México (UNAM), Boulevard Juriquilla 3001, Querétaro 76230, Mexico; ^2^Silvio O. Conte Center for Oxytocin and Social Cognition, Center for Translational Social Neuroscience, Department of Psychiatry and Behavioral Sciences, Yerkes National Primate Research Center, Emory University, 954 Gatewood Rd., Atlanta, GA 30329, USA; ^3^Escuela Nacional de Estudios Superiores, Unidad Juriquilla, UNAM, Mexico; ^4^Instituto Nacional de Perinatología Isidro Espinosa de los Reyes, Montes Urales 800, Col. Lomas Virreyes, Del. Miguel Hidalgo, Ciudad de México 11000, Mexico

## Abstract

*Microtus ochrogaster* is a rodent with a monogamous reproductive strategy characterized by strong pair bond formation after 6 h of mating. Here, we determine whether mating-induced pair bonding increases cell proliferation in the subventricular zone (SVZ), rostral migratory stream (RMS), and dentate gyrus (DG) of the hippocampus in male voles. Males were assigned to one of the four groups: (1) control: males were placed alone in a clean cage; (2) social exposure to a female (SE m/f): males that could see, hear, and smell a sexually receptive female but where physical contact was not possible, because the animals were separated by an acrylic screen with small holes; (3) social exposure to a male (SE m/m): same as group 2 but males were exposed to another male without physical contact; and (4) social cohabitation with mating (SCM): males that mated freely with a receptive female for 6 h. This procedure leads to pair bond formation. Groups 2 and 3 were controls for social interaction. Male prairie voles were injected with 5-bromo-2′-deoxyuridine (BrdU) during the behavioral tests and were sacrificed 48 h later. Brains were processed to identify the new cells (BrdU-positive) and neuron precursor cells (neuroblasts). Our principal findings are that in the dorsal region of the SVZ, SCM and SE m/f and m/m increase the percentage of neuron precursor cells. In the anterior region of the RMS, SE m/f decreases the percentage of neuron precursor cells, and in the medial region SE m/f and m/m decrease the number of new cells and neuron precursor cells. In the infrapyramidal blade of the subgranular zone of the DG, SE m/m and SCM increase the number of new neuron precursor cells and SE m/m increases the percentage of these neurons. Our data suggests that social interaction, as well as sexual stimulation, leads to pair bonding in male voles modulating cell proliferation and differentiation to neuronal precursor cells at the SVZ, RMS, and DG.

## 1. Introduction

Social monogamy is a reproductive strategy characterized by low levels of multiple paternity, biparental care of the offspring, low levels of infanticide, and cooperation [1]. *Microtus ochrogaster* (prairie vole) is a socially monogamous rodent species. When a female and a male cohabitate with mating for 6 h or cohabitate without mating for 24 h, they establish a strong pair bond. Thus, sexual activity accelerates this sociosexual behavior. A pair bond is characterized by mate guarding, cohabitation in the nest, sharing and defending their territory, and biparental care of the offspring [2]. These social behaviors favor stable family structures [3–6]. Once a pair bond is formed, it keeps prairie voles together for a long time, and when one of the voles dies, the survivor usually does not pair bond with a new mating partner [7, 8]. Because of these characteristics, the prairie vole is ideal to study the neurobiological mechanisms involved in pair bonding [9].

In rodents, social behaviors require the recognition of conspecifics through the olfactory system. In prairie voles, the integrity of the olfactory bulb (OB) is essential for the expression of social and sexual behavior. Males with bilateral bulbectomy (lesions in the main and accessory olfactory bulbs) decreased socialization time with their siblings, did not establish partner preference, and attacked the offspring, and only 9% of them mated. Females with the same lesion showed maternal care but reduced their social contacts with males and did not show a preference for their sexual partner, and only 17% of them exhibited behavioral estrus [10, 11]. These behavioral effects relay principally in the accessory olfactory bulb (AOB). Male prairie voles whose vomeronasal organ was removed spent less time sniffing females in comparison to control animals. After cohabiting with sexually receptive females for 8 weeks, male voles decreased their reproductive rate, as only 22% had offspring, while 75% of the males with a sham lesion produced progeny [12]. Female prairie voles lesioned in the vomeronasal organ did not show estrus behavior, and only 44% mated [13]. Moreover, when induced into estrus by the administration of estradiol, they mated but did not pair bond [14]. Anatomically, the OB and the hippocampus are connected through the entorhinal cortex and directly with specific cells of the ventral hippocampus projecting to the OB [15, 16]. The activity of both regions modulates odor recognition and olfactory learning and memory [17, 18]. The OB and hippocampus are structures that change their activity and functional organization in response to environmental and physiological stimulation (reviews in [19–24]).

Adult neurogenesis is a plastic change observed in most vertebrate species and has been involved in several physiological processes including sexual and maternal behavior, gestation, recognition of sexual partner and offspring, cognition, memory, learning, and brain repair [25–27]. New cells are continually produced in the subventricular zone (SVZ), rostral migratory stream (RMS), and subgranular zone (SGZ) of the dentate gyrus (DG) of the hippocampus [28]. New cells from the SVZ and RMS migrate and reach the OB, where they integrate into the granular and glomerular layers of the main olfactory bulb (MOB) and AOB [29, 30]. New cells from the SGZ migrate a short distance to the granule cell layer of the DG, and when they reach maturity, they send axons into the CA3 region of the hippocampus [31, 32].

Adult neurogenesis plays a fundamental role in sexual behavior and social and sexual partner recognition, where exposure to pheromones and sexual behavior modulates this plastic process. Thus, male mice in which adult neurogenesis was eliminated showed a decreased frequency and duration of mounting compared to control mice [33]. Similarly, in male rats, the intracerebroventricular infusion of the antimitotic drug cytosine arabinose (Ara-c) inhibited adult neurogenesis. Rats that had been subject to this treatment showed a decrease in intromission frequency and copulatory efficiency (total number of intromissions/sum of intromissions and mount frequency), and males were not able to ejaculate [34].

In rodents, termination of early pregnancy can occur if the female is exposed to pheromones of a male that is not the father of her carried embryos, a phenomenon known as the Bruce effect [35]. Oboti and collaborators demonstrated that the administration of Ara-c induces a strong impairment of sexual partner recognition. Ara-c administrated to female mice during early pregnancy, in the presence of their sexual partner's pheromones, triggered a neuroendocrine reflex that leads to pregnancy block [36].

Sexual behavior modulates adult neurogenesis in male rodents. Male rats that mate and ejaculate one to three times showed an increase in the number of new cells that migrate to the granular layer of the AOB [37]. Males that ejaculate three times showed an increase in the number of new cells that survive, and a high percentage of those cells differentiate into mature neurons in the granular layer of the AOB [38]. In sexually naïve male mice, the first sexual experience increased the percentage of new cells that differentiate into neurons in the glomerular cell layer of the MOB compared with males exposed to a sexually receptive female and control males [39].

Rats that mate once for 30 min increases cell proliferation in the DG of the hippocampus, and repeated sexual intercourse (30 min for 14 consecutive days) increases proliferation and survival of the new cells in this neuronal region [40]. The physiological importance of neurogenesis in reproductive behaviors has also been addressed in prairie voles. In females, it has been shown that estrus induction by exposure to non-family-related males increases cell proliferation in the RMS by 90%, and 80% of the cells commit to the neuronal lineage [41]. Cohabitation with a male increases cell proliferation in the amygdala and the hypothalamus but has no effect in the SVZ [42].

To our knowledge, there are no studies on male prairie voles evaluating whether sexual behavior that leads to pair bonding or social exposure to a male or female induces cell proliferation in the SVZ, RMS, and SGZ of the DG of the hippocampus. Since sexual behavior and pheromone exposure modulate adult neurogenesis in mice and rats, and the new cells play a fundamental role in sexual behavior and social and sexual partner recognition, we hypothesize that in male voles, sexual stimulation could increase cell proliferation in these germinal areas.

To evaluate the sociosexual effect of the female, males were exposed to a female for 6 h, a procedure that does not induce pair bonding because at least 24 h of cohabitation is required to induce this social behavior [43]. As a control for social interaction, a group of males exposed to other males was evaluated. Males that were housed alone were included as a control group for sexual and social interactions.

## 2. Methods

### 2.1. Animal Handling

Adult sexually mature male and female voles (three to four months old) were housed in a 14 h light : 10 h dark cycle with food (rabbit diet high fiber 5326 (LabDiet), oats, and sunflower seeds) and water available *ad libitum*. The prairie voles derived from a colony at Emory University that originated from wild stock from Illinois, USA. The mating pairs were formed by one male and one female of different families. After weaning, prairie voles (4/5) of the same sex and age were housed together. All experiments were performed in accordance with the “Reglamento de la Ley General de Salud en Materia de Investigación para la Salud” of the Mexican Health Ministry, which is based on NIH guidelines for the use and care of animals in research. The experiments were approved by the Animal Care Committee of the Instituto de Neurobiología and by the Ethics Committee of the Instituto Nacional de Perinatología.

### 2.2. Behavioral Testing

Adult female voles were ovariectomized, and sexual receptivity was induced by daily subcutaneous injections of estradiol benzoate (EB, 0.5 *μ*g/vole, Sigma-Aldrich) for four days before the behavioral test. This procedure consistently induces sexual receptivity [24, 44, 45].

**Figure 1:**
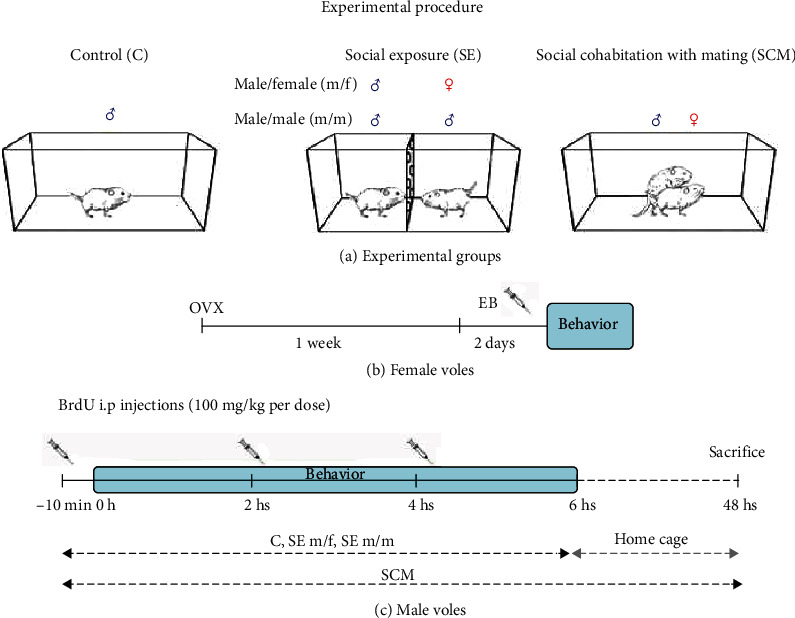
Schematic diagram of the experimental procedure. (a) Experimental conditions for the control (C), social exposure to a female (SE m/f), social exposure to a male (SE m/m), and social cohabitation with mating (SCM) groups. (b) Adult female voles were bilaterally ovariectomized, and after one week of recovery, they were treated with estradiol benzoate to induce sexual receptivity. (c) To visualize the new cells, adult male voles were injected with 5-bromo-2′-deoxyuridine (BrdU) three times during the sexual behavior tests and were sacrificed 48 h after completing the tests. OVX: bilateral ovariectomy; EB: estradiol benzoate.

Twenty-eight adult males were randomly distributed into the following four groups: (1) control (C; *N* = 7): animals were placed alone in a clean acrylic cage; (2) social exposure to a female (SE m/f; *N* = 7): male voles were placed in acrylic cages divided into two compartments by a plastic screen with small holes. In this condition, they were able to hear, smell, and see the receptive female placed in the other compartment but had no physical contact with her; (3) social exposure to a male (SE m/m; *N* = 7): same as the SE m/f group but the stimulus was another unfamiliar male; and (4) social cohabitation with mating (SCM; *N* = 7): male voles were placed in an acrylic cage in which a sexually receptive female was introduced 15 min before the male. In this condition, the male was free to mate with his female (Figures 1(a) and 1(b)). Behavioral testing lasted 6 h. For the SCM, it has been reported that this time of mating consistently induces pair bonding [43]. At the end of the 6 h, C and SE m/f males were put back in their home cages with their respective group cage mates. This was necessary because it has been reported that 24 h of cohabitation with a female without mating induce pair bonding and social isolation can induce stress and decreases adult neurogenesis [43, 46]. This allowed us to differentiate the pair bond induced by mating from that induced by long cohabitation periods. SCM and SE m/m voles remained with their conspecific until the day of sacrifice. SE m/m voles were a control for general social interaction. The following male sexual behavior parameters were recorded in the first 2 h of the test for the SCM: number and latency of mounts, intromissions and ejaculations, and postejaculatory interval.

To label new cells, male voles were injected with the DNA synthesis marker 5-bromo-2′-deoxyuridine (BrdU) dissolved in 0.9% NaCl. Subjects were injected 100 mg/kg i.p. every 2 h (3 times total; Figure 1(c)) with the first injection at 10 min before the beginning of the test. BrdU is rapidly degraded *in vivo*, and its bioavailability ranges from 15 min to 2 h in several tissues [47–49]. This allowed us to only label cells that were in the S phase of the cell cycle during the behavioral test.

Forty-eight hours later, male voles were anesthetized with an overdose of pentobarbital (6.3 mg/vole, Cheminova) and perfused intracardially with 0.1 M phosphate-buffered saline (PBS) followed by 4% paraformaldehyde (PFA, Sigma-Aldrich) dissolved in PBS. Vole brains were removed, postfixed by immersion in 4% PFA-PBS for 1 h, and stored in 30% sucrose (J.T. Baker)-PBS solution at 4°C.

### 2.3. Immunostaining Assay

Thirty-micron-thick sagittal brain sections were obtained using a Leica CM1850 cryostat. Nine sagittal sections with the SVZ and RMS were obtained, and three sections were analyzed per prairie vole. Thus, the interval between slices was 60 *μ*m. For the DG, three sections were evaluated bilaterally. Brain sections of the SVZ, RMS, and DG were matched neuroanatomically across all animals. For double immunolabeling of BrdU and the neuron precursor cells marker doublecortin (DCX), free-floating brain sections were washed three times for 10 min each in Tris-buffered saline (TBS, pH 7.6, J.T. Baker). Then, brain sections were incubated with 2 N HCl (J.T. Baker) at 37°C for 30 min and subsequently were rinsed in 0.1 M citrate buffer (Sigma-Aldrich) pH 6 for 30 min. Sections were washed three times for 10 min each in washing buffer (TBS with 0.05% Tween-20) and incubated in 1% H_2_O_2_ (J.T. Baker)-TBS solution for 30 min at room temperature (RT). Nonspecific labeling was blocked using a blocking solution (TBS with 1% *w*/*v* bovine serum albumin fraction V A9418 (Sigma-Aldrich) and 3% *v*/*v* normal goat serum) for 30 min RT. Immunodetection was performed using the following primary antibodies diluted in TBS: rat anti-BrdU OBT0030 (Serotec, 1 : 800) and guinea pig anti-DCX AB2253 (Millipore, 1 : 4000), incubated overnight at 4°C. Both primary antibodies were added at the same time. Slides were washed three times for 10 min each with washing buffer and incubated with the secondary antibodies diluted in TBS, biotinylated anti-rat BA-9400 (Vector Laboratories, 1 : 2000) and anti-guinea pig Alexa Fluor 488 (Molecular Probes Invitrogen, 1 : 2000) for 1.5 h at RT. After three washes, sections were incubated with streptavidin conjugated with Cy3 (Jackson ImmunoResearch, 1 : 1000) for 1.5 h at RT to visualize BrdU-specific immunolabeling. Finally, brain slides were mounted with Aqua-Poly/Mount (Polysciences) and stored in darkness at 4°C until microscope analysis. Negative controls for specific immunolabeling were obtained in slices from voles not injected with BrdU and without primary antibodies; in negative controls, no positive cells were detected.

### 2.4. Cell Counting

**Figure 2:**
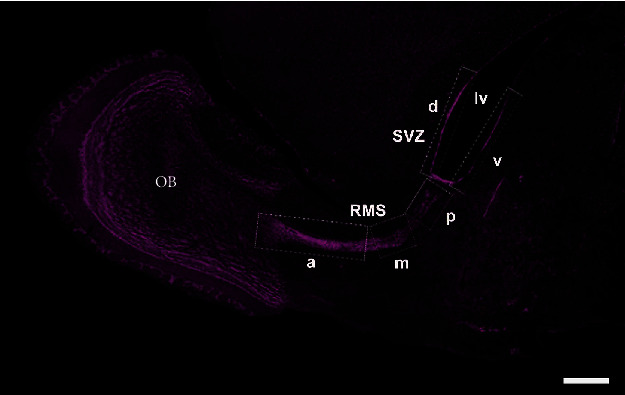
Sagittal section of an adult male vole brain stained with the nuclear marker Hoechst (magenta pseudocolored). White scattered rectangles indicate the subdivision of the subventricular zone (SVZ) and rostral migratory stream (RMS). SVZ subareas: d: dorsal; v: ventral. RMS subareas: a: anterior; m: medial; p: posterior. lv: lateral ventricle. OB: olfactory bulb. Photomicrographs were taken at 20x. Scale bar: 500 *μ*m.

Sagittal brain sections processed for immunostaining using anti-BrdU and anti-DCX primary antibodies were examined using a Zeiss AX10 confocal microscope, by means of a 20x objective (total magnification was 200x (20x objective × 10 projection ocular magnification)), and digitalized with MacBioPhotonics ImageJ. Photomicrographs with 4-6 planes in the *z*-axis were taken. Some animals were excluded from the analysis if three well-preserved and complete sections were not obtained from a particular brain region. Cell count was performed in one of the *z*-axis stacks, usually the central one, containing the better fluorescence signal. To determine double label, cells were followed in all *z*-axis stacks. Filters of size (50-infinity) and circularity (0.50-1.00) were applied using the particle analysis tool of ImageJ, which allows us to exclude unspecific labeling. The new cells that integrate to the olfactory bulb are born in different regions of the SVZ and RMS (Alvarez Buylla 1993) [50–52]; therefore, the SVZ was divided into the dorsal and ventral regions, whereas the RMS was divided into the anterior, medial, and posterior regions (Figure 2) as previously described [53]. The DG of the hippocampus was divided into the suprapyramidal and infrapyramidal blades [54]. The hilus region was only included in the analysis of total cell proliferation. The number of BrdU-positive cells was evaluated, to determine if social or sexual stimuli increased cell proliferation. The percentage of BrdU/DCX-positive cells was calculated as the number of BrdU/DCX-positive cells divided by the number of BrdU-positive cells and multiplied by 100. This parameter denotes whether the experimental manipulation favors cell fate into the neuronal linage.

### 2.5. Statistical Analysis

Data, expressed as means plus SEM, were analyzed using Prism 5 (GraphPad Software, Inc.) and SigmaPlot 14.0. Cell counts were averaged from three sections, for each brain area, and means were used for data analysis. Normal distribution was evaluated with the normality test (Shapiro-Wilk) and equal variance test. Since our data show a normal distribution, statistical differences were determined by one-way ANOVA followed by Bonferroni's post hoc tests. A value of *p* < 0.05 was considered significant. The effect size in ANOVA was evaluated with the eta squared (*η*^2^), calculated as SS (between groups)/SS (total). SS is the sum of squares.

## 3. Results

### 3.1. Behavioral Test

Sexual behavior parameters in the SCM group were recorded during the first 2 of the 6 h behavioral test. The sexual behavior displayed by the males (Table 1) is consistent with previous published observations [24], indicating that BrdU administration did not affect sexual behavior in adult male voles.

### 3.2. Proliferation Rate and Neuron Precursor Positive Cells

#### 3.2.1. Subventricular Zone

**Figure 3:**
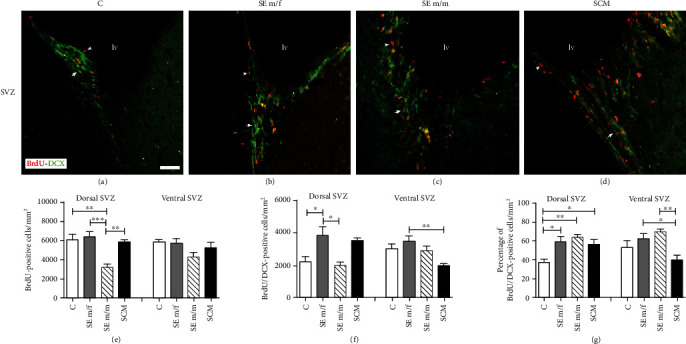
Representative photomicrographs of double immunolabeling for BrdU (red) and DCX (green) in the dorsal SVZ in the control (C; (a)), social exposure to a female (SE m/f; (b)), social exposure to a male (SE m/m; (c)), and social cohabitation with mating (SCM; (d)). White arrows indicate BrdU-positive/DCX-positive cells (organized in chain arrangements), and white arrowheads indicate BrdU-positive/DCX- negative cells. Photomicrographs were taken at 20x. Scale bar: 100 *μ*m. Data analysis of cell proliferation, total BrdU/DCX-positive cells, and percentage of BrdU/DCX-positive cells in the C, SE m/f, SE m/m, and SCM animals are shown in (e–g). lv: lateral ventricle. Data were analyzed by one-way ANOVA followed by Bonferroni's post hoc tests. ^∗^*p* < 0.05 and ^∗∗^*p* < 0.01. Results of ANOVA analyses are provided in the text. Dorsal SVZ: C (*n* = 7), SE m/f (*n* = 7), SE m/m (*n* = 6), and SCM (*n* = 6); ventral SVZ: C (*n* = 5), SE m/f (*n* = 7), SE m/m (*n* = 6), and SCM (*n* = 6).

*(1) Dorsal Region*. Representative photomicrographs of the BrdU-positive and BrdU/DCX-positive cells are shown in Figures 3(a)–3(d). Significant differences between groups were found in the number of BrdU-positive cells in the dorsal region (*F*_(3, 25)_ = 9.7, *p* < 0.001, *η*^2^ = 0.6; Figure 3(e)). The number of BrdU-positive cells was reduced in the SE m/m group in comparison to the other groups (different from C (*p* = 0.002), SE m/f (*p* < 0.001), and SCM (*p* = 0.003)). Significant differences in the number of neuron precursor cells (BrdU/DCX-positive cells) were also found (*F*_(3, 25)_ = 5.6, *p* = 0.005, *η*^2^ = 0.4; Figure 3(f)). Post hoc tests showed that the SE m/f group had a higher number of BrdU/DCX cells in comparison to the C (*p* = 0.03) and SE m/m (*p* = 0.012) groups. The analysis of the percentage of BrdU/DCX-positive cells revealed significant differences between groups (*F*_(3, 25)_ = 6.6, *p* = 0.002, *η*^2^ = 0.5; Figure 3(g)). Post hoc tests demonstrate that all groups had an increased percentage of BrdU/DCX-positive cells in comparison to C voles (SE m/f (*p* = 0.011), SE m/m (*p* = 0.004), and SCM (*p* < 0.05)).

*(2) Ventral Region*. No significant differences were found between groups in the number of BrdU-positive cells in the ventral region of the SVZ (*F*_(3, 23)_ = 1.9, *p* = 0.2, *η*^2^ = 0.2; Figure 3(e)). Significant differences between groups were also found in the number of BrdU/DCX-positive cells (*F*_(3, 23)_ = 5.3, *p* = 0.008, *η*^2^ = 0.4; Figure 3(f)). Post hoc tests demonstrated that SE m/f males had more neuron precursor cells than voles from the SCM group (*p* = 0.005). Significant differences between groups were found in the percentage of BrdU/DCX-positive cells (*F*_(3, 23)_ = 5.8, *p* = 0.005, *η*^2^ = 0.5; Figure 3(g)). The SE m/m (*p* = 0.005) and m/f (*p* = 0.037) groups had a higher percentage of neuron precursor cells than SCM males.

#### 3.2.2. Rostral Migratory Stream

**Figure 4:**
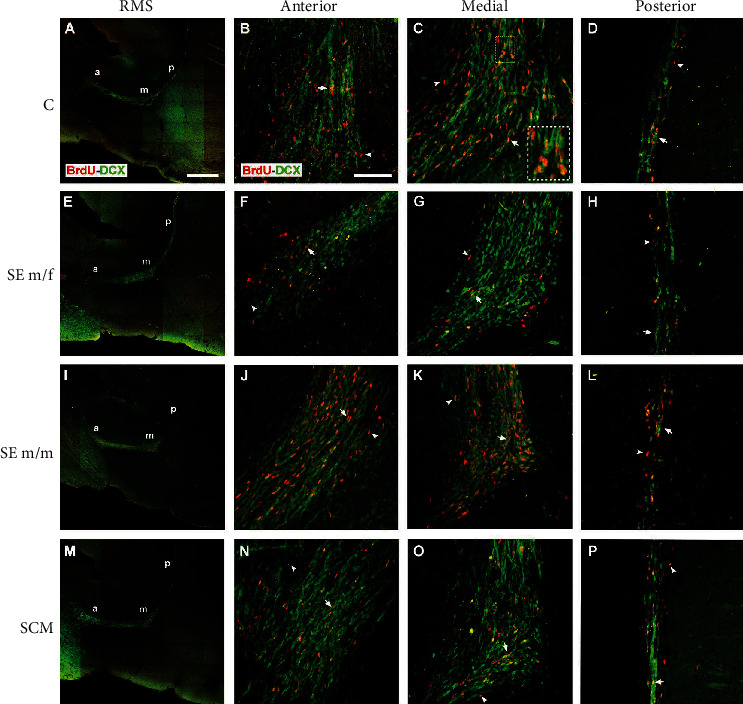
Representative photomicrographs of double immunolabeling for BrdU and DCX in the RMS subareas (red/green; (a, e, i, m); photomicrographs were taken at 20x, scale bar: 500 *μ*m). Representative photomicrographs (20x, scale bar: 100 *μ*m) of the anterior, medial, and posterior subareas of the RMS. Control (C; (a–d)), social exposure to a female (SE m/f; (e–h)), social exposure to a male (SE m/m; (i–l)), and social cohabitation with mating (SCM; (m–p)). A scattered square (panel C) represents enlargements of the inserts. White arrows indicate BrdU-positive/DCX-positive cells organized in chain arrangements, and arrowheads point to BrdU-positive/DCX-negative cells. a: anterior; m: medial; p: posterior.

**Figure 5:**
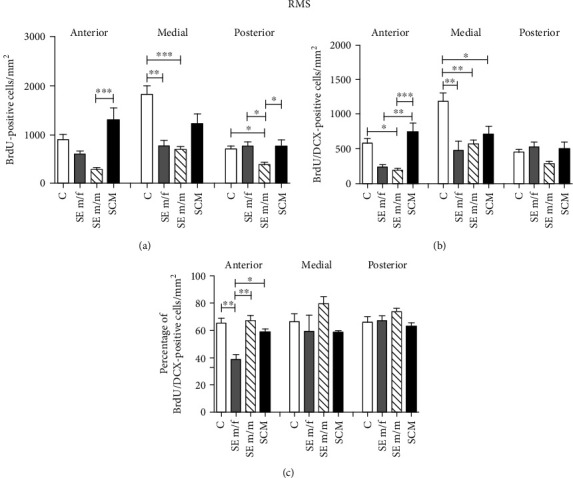
Cell proliferation, total BrdU/DCX-positive cells, and percentage of BrdU/DCX-positive cells in the RMS (a–c) for the control (C), social exposure to a female (SE m/f), social exposure to a male (SE m/m), and social cohabitation with mating (SCM) male voles. One-way ANOVA followed by Bonferroni's post hoc tests. ^∗^*p* < 0.05 and ^∗∗^*p* < 0.01. Results of ANOVA analyses are provided in the text. Anterior RMS: C (*n* = 6), SE m/f (*n* = 3), SE m/m (*n* = 6), and SCM (*n* = 6); medial RMS: C (*n* = 7), SE m/f (*n* =  4), SE m/m (*n* = 5), and SCM (*n* = 6); and posterior RMS: C (*n* = 7), SE m/f (*n* = 6), SE m/m (*n* = 6), and SCM (*n* = 5).

*(1) Anterior Region*. Representative photomicrographs of BrdU and BrdU/DCX-positive cells in the RMS are shown in Figure 4. Significant differences were found in the number of BrdU-positive cells between groups in the anterior region of the RMS (*F*_(3, 20)_ = 8, *p* = 0.002, *η*^2^ = 0.6; Figure 5(a)). Post hoc tests demonstrate that the SCM group had more BrdU label cells than the SE m/m subjects (*p* = 0.001).

Significant differences were found in the number of BrdU/DCX-positive cells (*F*_(3, 20)_ = 11, *p* < 0.001, *η*^2^ = 0.7; Figure 5(b)); the SCM voles showed an increase in label cells in comparison to the SE m/f (*p* = 0.008) and SE m/m groups (*p* < 0.001). The SE m/m group also had fewer label cells in comparison to the C group (*p* = 0.013). Significant differences were also found in the percentage of BrdU/DCX-positive cells (*F*_(3, 20)_ = 7, *p* = 0.003, *η*^2^ = 0.6; Figure 5(c)). Post hoc tests demonstrate that the SE m/f males showed a decrease in the percentage of new neuron precursor cells in comparison to the other groups (C (*p* = 0.006), SE m/m (*p* = 0.003), and SCM (*p* = 0.047)).

*(2) Medial Region*. In the medial region, significant differences were found (*F*_(3, 21)_ = 10, *p* < 0.001, *η*^2^ = 0.6; Figure 5(a)) in the number of BrdU-positive cells; post hoc tests demonstrated that the SE m/f (*p* = 0.003) and m/m (*p* < 0.001) groups showed a decrease in the number of BrdU-positive cells in comparison to C voles. Significant differences were also found in the number of BrdU/DCX-positive cells (*F*_(3, 21)_ = 8.2, *p* = 0.001, *η*^2^ = 0.6; Figure 5(b)); the SE m/f (*p* = 0.003), SE m/m (*p* = 0.006), and SCM (*p* = 0.03) males showed a significant reduction in the number of BrdU/DCX-positive cells in comparison to C males. There were no significant differences in the percentage of BrdU/DCX-positive cells between groups (*F*_(3, 21)_ = 2.4, *p* = 0.1, *η*^2^ = 0.3; Figure 5(c)).

*(3) Posterior Region*. Significant differences were found in the posterior region of the RMS in the number of BrdU-positive cells (*F*_(3, 24)_ = 5, *p* = 0.009, *η*^2^ = 0.4; Figure 5(a)). Post hoc tests demonstrated that the SE m/m group had a reduced number of label cells in comparison to the other groups (C (*p* = 0.042), SE m/f (*p* = 0.018), and SCM (*p* = 0.03)). No significant differences between groups were found in the number (*F*_(3, 24)_ = 3.1, *p* = 0.05, *η*^2^ = 0.3) and percentage of BrdU/DCX-positive cells (*F*_(3, 24)_ = 1.2, *p* = 0.3, *η*^2^ = 0.2; Figures 5(b) and 5(c)).

#### 3.2.3. Dentate Gyrus of the Hippocampus

**Figure 6:**
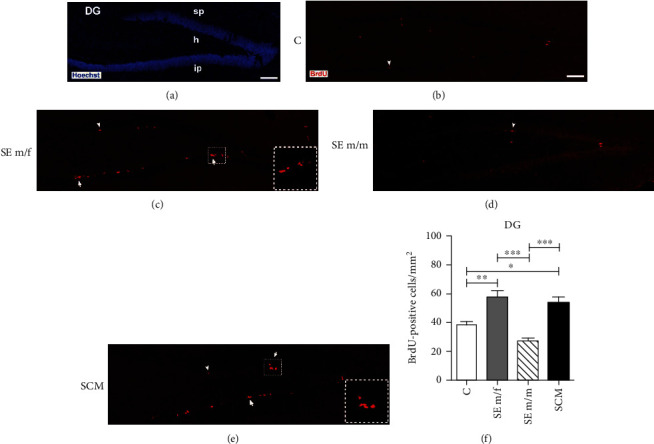
Photomicrographs of nuclei stained with Hoechst showing DG of the hippocampus in the male vole brain (a). Immunolabeling for BrdU in the DG from the control (C), social exposure to a female (SE m/f), social exposure to a male (SE m/m), and social cohabitation with mating (SCM) animals, respectively (b–e). White arrowheads point to single BrdU-positive cells, and white arrows indicate clusters of BrdU-positive cells. Scattered squares represent enlargements of the inserts at 20x, which show clustering of proliferating cells in the DG from the SE m/f and SCM voles. Data analysis for cell proliferation in the whole DG, including the hilus, for the C, SE m/f, SE m/m, and SCM animals (f). Data were analyzed with one-way ANOVA followed by Bonferroni's post hoc tests. sp: suprapyramidal blade; ip: infrapyramidal blade; h: hilus. 20x. Scale bar: 100 *μ*m. ^∗^*p* < 0.05, ^∗∗^*p* < 0.01, and ^∗∗∗^*p* < 0.001. Results of ANOVA analyses are provided in the text. C (*n* = 8), SE m/f (*n* = 10), SE m/m (*n* = 5), and SCM (*n* = 9).

**Figure 7:**
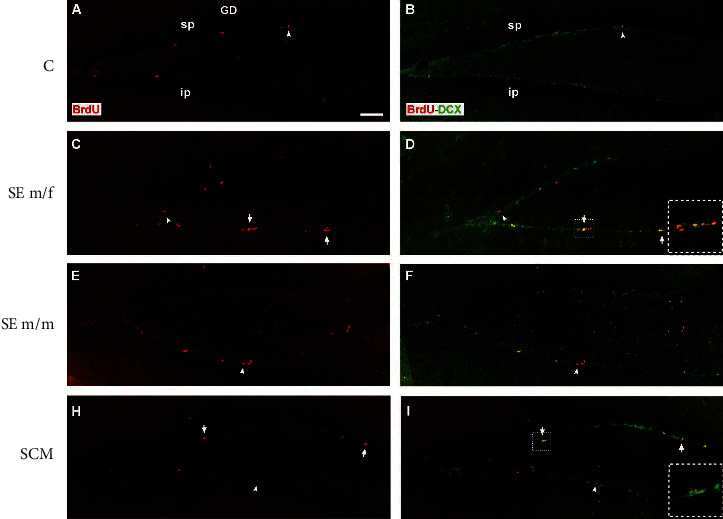
Representative photomicrographs of immunolabeling for BrdU (red; (a, c, e, h)) and double immunolabeling for BrdU/DCX (red/green; (b, d, f, i)) from the DG of the control (C), social exposure to a female (SE m/f), social exposure to a male (SE m/m), and social cohabitation with mating (SCM) animals. White arrowheads point to single BrdU-positive cells, and white arrows indicate clusters of BrdU/DCX-positive cells. Scattered squares: enlargements of the inserts at 20x, which show clustering of proliferating neuroblasts observed in the DG from the SE m/f and SCM voles. sp: suprapyramidal blade; ip: infrapyramidal blade. 20x. Scale bar: 100 *μ*m.

The number of BrdU-positive cells was evaluated in the suprapyramidal and infrapyramidal blades of the DG and the hilus (Figures 6(a)–6(e) and 7). ANOVA tests demonstrate significant differences between groups in the number of BrdU-positive cells (*F*_(3, 31)_ = 11.4, *p* < 0.001, *η*^2^ = 0.6; Figure 6(f)). Post hoc tests demonstrate that the SE m/f and SCM voles showed an increase in the number of BrdU-positive cells in comparison to the C (*p* = 0.005 and *p* = 0.04, respectively) and SE m/m males (*p* < 0.001 and *p* = 0.001, respectively).

**Figure 8:**
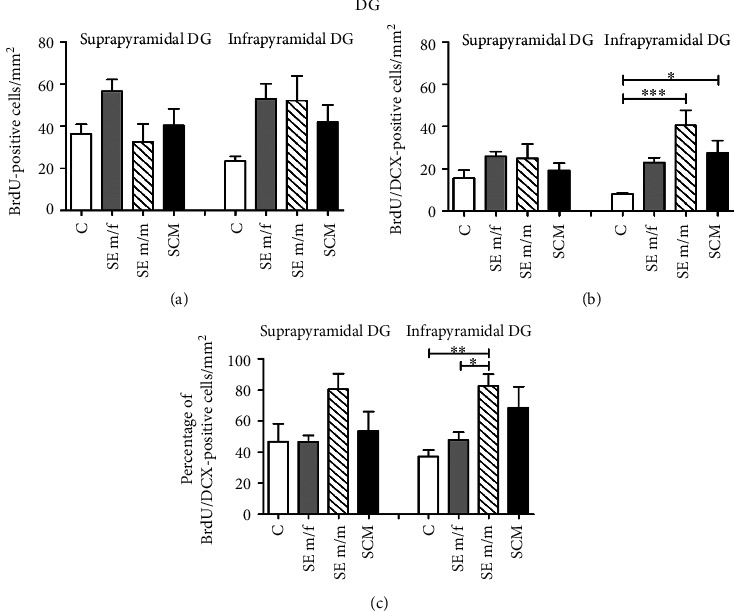
Cell proliferation, total BrdU/DCX-positive cells, and percentage of BrdU/DCX-positive cells (a–c) for the control (C), social exposure to a female (SE m/f), social exposure to a male (SE m/m), and social cohabitation with mating (SCM) male voles. Data were analyzed by one-way ANOVA followed by Bonferroni's post hoc test. ^∗^*p* < 0.05, ^∗∗∗^*p* < 0.001. Results of ANOVA analyses are provided in the text. Suprapyramidal blade of the DG: C (*n* = 7), SE m/f (*n* = 7), SE m/m (*n* = 6), and SCM (*n* = 5); infrapyramidal blade of the DG: C (*n* = 5), SE m/f (*n* = 6), SE m/m (*n* = 5), and SCM (*n* = 5).

*(1) Suprapyramidal Blade of the DG of the Hippocampus*. In the suprapyramidal blade, no significant differences were found between groups in the number of BrdU-positive cells (*F*_(3, 24)_ = 2.9, *p* = 0.06, *η*^2^ = 0.3) and in the number (*F*_(3, 24)_ = 1.3, *p* = 0.3, *η*^2^ = 0.2) or percentage of BrdU/DCX-positive cells (*F*_(3, 24)_ = 2.8, *p* = 0.07, *η*^2^ = 0.3) (Figures 8(a)–8(c)).

*(2) Infrapyramidal Blade of the DG of the Hippocampus*. No significant differences were found in the number of BrdU cells in the infrapyramidal blade of the DG of the hippocampus (*F*_(3, 20)_ = 3, *p* = 0.06, *η*^2^ = 0.4; Figures 7 and 8(a)). Significant differences were found in the number of BrdU/DCX-positive cells (*F*_(3, 20)_ = 8.3, *p* = 0.001, *η*^2^ = 0.6; Figure 8(b)). Post hoc tests demonstrated that the SE m/m (*p* < 0.001) and SCM (*p* < 0.05) males showed an increase in new neuron precursor cells in comparison to C voles. Significant differences were found in the percentage of BrdU/DCX-positive cells (*F*_(3, 20)_ = 6.2, *p* = 0.005, *η*^2^ = 0.5; Figure 8(c)); post hoc tests demonstrated that the SE m/m group showed an increase in the number of label cells in comparison to the C (*p* = 0.007) and SE m/f (*p* = 0.038) voles.

## 4. Discussion

### 4.1. Subventricular Zone

Our data showed that mating, exposure to a female without physical contact, and control conditions (isolation of the male) induced an increased proliferation in the dorsal region of the SVZ compared to males exposed to another male. Voles exposed to a sexually receptive female had more new neuron precursor cells in the dorsal region of the SVZ in comparison to controls and males exposed to another male, whereas males that were exposed to a female or male and voles that mated for 6 h showed an increase in the percentage of neuron precursor cells in comparison to control voles in the dorsal SVZ. Thus, social and mating stimulation favors the differentiation to the neuronal lineage. The dorsal SVZ generates superficial dopaminergic periglomerular cells and granular OB neurons [55–57]. Thus, this increase in the percentage of neuron precursor cells in the dorsal region in voles that mate or were exposed to a conspecific can increase the number of new neurons that migrate into the granular and glomerular layers of the OB.

The ventral SVZ generates calbindin periglomerular interneurons and deep granular OB neurons [55, 56]. Our data demonstrate that in the ventral region of the SVZ, male voles exposed to a sexually receptive female show an increase in the number and percentage of neuron precursor cells in comparison to voles that mated, suggesting that voles exposed to a receptive female can generate more calbindin periglomerular and granular neurons in the OB.

These new cells generated by sociosexual stimulation can be integrated into the OB and eventually can be involved in recognition, discrimination, and learning of a conspecific odor [36, 58, 59]. Learning and recognition of the olfactory signature of a conspecific are fundamental in social affiliation and pair bonding in voles [10, 11].

Our data from the dorsal region of the SVZ agree with the results of the studies on female prairie voles where sexual cohabitation with mating increased cell proliferation in the SVZ compared to females exposed to another female, but no differences were found when females that had experienced sexual cohabitation with mating were compared to isolated voles [60].

Our results demonstrated that social and sexual exposure as well as mating increases the percentage of new neuron precursor cells in the dorsal SVZ in comparison to control males that were isolated for six hours. Previous studies demonstrated that social isolation for six weeks decreases cell proliferation, survival, and neuronal differentiation in limbic brain areas in female prairie voles [46]. Therefore, the decrease in the percentage of new neuron precursor cells in the dorsal SVZ can be due to social isolation. In female voles, mating does not favor the differentiation into neuron precursor cells in the SVZ [60]. In polygamous female rats, exposure to a male, but not mating, increases the percentage of new neuron precursor cells in the SVZ [53].

### 4.2. Rostral Migratory Stream

The RMS is primarily recognized as a migratory pathway, and its proliferative role is less recognized; proliferation is slower in the RMS than in the SVZ, generating most of the olfactory bulb interneuron subtypes calretinin granular and periglomerular cells [61]. Cell proliferation in the RMS is higher in the posterior and medial regions than in the anterior part [61, 62]. The medial and anterior regions of the RMS show a higher glial proliferation than the posterior region [63]. Thus, sexual activity, maternal care, and hormones modulate cell proliferation differently in each of the RMS subregions [41, 53, 64].

Our results demonstrate that social cohabitation with mating is followed by the increase of the number of new cells in the anterior region of the RMS in comparison to social exposure to a male. Mating and control voles had a higher number of neuron precursor cells in comparison to males exposed to a conspecific. Males exposed to a female showed a decrease in the percentage of new neurons. Data from the medial region of the RMS demonstrate that social exposure to a male or female decreases the number of new cells, and social exposure and mating decrease the number of neuron precursor cells, but not their percentage. The RMS medial region produces calretinin granule cells and periglomerular cells [51]; thus, a decrease in cell proliferation in this region can lead a reduction in calretinin granule and periglomerular cells in the olfactory bulb. Exposure to a male leads to a decrease in the number of new cells in the posterior RMS but does not affect neuronal proliferation. Our data suggest that, in male voles, social exposure to a male or female decreases cell proliferation, and exposure to a receptive female decreases neuronal phenotype determination in the RMS. Smith and coworkers demonstrated, in female voles, that estrus induction by exposure to a non-family-related male increases proliferation in the RMS in comparison to females exposed to their sister or a female from an unrelated litter. However, females exposed to a male did not increase the percentage of new cells that differentiated into neurons [41]. Similarly, in female rats, mating increases cell proliferation in the RMS but has no effect on cellular specification.

### 4.3. Dentate Gyrus of the Hippocampus

The DG of the hippocampus receives multiple sensory inputs, including vestibular, olfactory, visual, auditory, and somatosensory inputs, from the perirhinal and lateral entorhinal cortices and shares connections with the nucleus accumbens, amygdala, hypothalamus, and OB [15, 65]. The hippocampus is involved in the formation of social memory and social recognition [66]. In the present study, we found that social exposure to a female and cohabitation with mating increase the number of new cells in the DG and hilus of the hippocampus in comparison to control voles. Interestingly, social exposure to a male decreases cell proliferation in comparison to males that mated or were exposed to a female. When we analyzed cell proliferation in the infrapyramidal blade of the hippocampus, our data showed that exposure to a male and mating increase the number and exposure to a male increases the percentage of new neuron precursor cells in this region. No significant differences were found in the suprapyramidal blade of the DG.

This increase in neurogenesis in the infrapyramidal blade of the DG may be involved in partner memory formation in male voles, supporting the idea that olfaction-related memory process takes place in the hippocampus. These results are in agreement with previous observations on Golden hamsters (*Mesocricetus auratus*) showing that the presence of a familiar male increases the neuronal activity of the DG [67]. In addition, in male rats, repeated sexual experience (4 mating tests) with novel sexual partners in each test decreases neurogenesis in the DG. This decrease is not observed in males that mate repeatedly with the same sexual partner and in control males [68]. Mating induces cell proliferation in the DG in other mammals. Sexual stimulation increases cell proliferation in the DG of the hippocampus in male rats, too [40]. Similar results were obtained for the naked mole-rat (*Heterocephalus glaber*), a eusocial mammal in which only one female breeds with one or three males and the other members of the colony are subordinated and do not reproduce. Subordinates can reproduce after the death/removal of the breeder or separation from the colony [69]. Subordinates removed from the colony and housed with opposite sex animals show an increase in cell proliferation in the ventral DG in comparison to colony-housed subordinates and an increase in the number of neuron precursor cells in the ventral region of the DG in comparison to subordinates housed with their same sex. Cohabitation with mating effects over cell proliferation is specific to the ventral region, since no significant differences were found in the dorsal DG [70]. However, female prairie voles in cohabitation with mating or in cohabitation with another female did not show increased cell proliferation in the DG or in the percentage of new cells that differentiate into neurons [60].

Sexually naïve, single male voles are highly social and display affiliative behavior toward novel conspecifics [7]. They can discriminate between males but not females, probably because identifying other males is more meaningful at this stage [71, 72]. Our data demonstrate that male voles exposed to other males showed an increase in the percentage of neuron precursor cells in the dorsal SVZ and infrapyramidal blade of the DG; these new neurons could be involved in the development of the memory of the familiar male identity. Sexually naïve (single) male prairie voles are not able to discriminate between two sexually naïve females. However, male voles that cohabitate and mate with females for 24 h forming pair bonding can discriminate between two unfamiliar sexually naïve females [71]. Thus, pair-bonded males exhibited social recognition of females, but single males do not. We found that males that mate with a female increase the percentage of new neuron precursor cells in the dorsal SVZ and new cells in the DG. Further studies are needed to determine if these new neurons can be involved in sexual partner memory and social recognition.

### 4.4. Factors That Might Modulate Cell Proliferation Induced by Sociosexual Behaviors

The mechanisms involved in the increase of cell proliferation and neuronal differentiation induced by sexual behavior and exposure to a member of the opposite sex are not well understood. Opioids modulate adult neurogenesis; administration of the opioid antagonist naloxone blocked the increase of new cells and neurons generated after mating stimulation in female rats [73]. The SVZ expresses *κ*-opioid and *β*-endorphin receptors. The administration of the *κ*-opioid receptor agonist, ICI 204448, as well as the endogenous opioid ligand *β*-endorphin increases nestin quiescent neuronal stem cells in culture. *In vivo*, ICI 204448 increases cell proliferation in the SVZ without modifying the expression of neuroblasts [74].

Oxytocin may also be involved in the increase in cell proliferation. Oxytocin increases neurogenesis in the DG and modulates the survival and maturation of new dentate granule cells via oxytocin receptors expressed in CA3 pyramidal neurons in male mice [75]. Oxytocin administration to male rats increases social interactions (sniffing, following, crawling, social play, and grooming) and proliferation of new cells, but not cell differentiation in the hippocampus [76].

Dopamine (D) also modulates neurogenesis and sexual behavior. Activation of D2 and D3 receptors increases the *in vitro* proliferation of neurospheres from the SVZ and increases the differentiation into glial and neuron precursor cells in mice [77]. The D3 receptor is expressed in progenitor cells in the SVZ, and its activation increases cell proliferation. Administration of a D3 antagonist decreases the number of new cells in the RMS and periglomerular neurons in the OB [78, 79]. Neuronal stem cells in the SVZ receive nigral dopaminergic projections and express D2 and D3 receptors. Further studies are needed to determine if social exposure and social cohabitation with mating modulate proliferation through mechanisms mediated by opioids, oxytocin, or dopamine.

We hypothesize that neurogenesis leading to new neurons in the olfactory bulb and hippocampus may contribute to the formation of long-term memories or preferential responsiveness to partner odors which may play an important role in pair bond formation and social interactions. Further studies are needed to determine if in male voles social cohabitation with mating or the exposure to a conspecific increases cell survival in the olfactory bulbs and other brain regions.

## Figures and Tables

**Table 1:** Sexual behavioral parameters in the social cohabitation with mating group, where subjects could mate freely. The data was collected during the first 2 h (*n* = 7).

Sexual parameter	Range	Max	Min	Median	25%	75%
MN	328	343	15	18	16	35
IN	25	49	24	25	24	41
EN	1	3	2	2	2	2
ML	188	312	124	258	147	300
IL	546	816	270	408	369	609.5
EL	1300.2	1870.2	570	1040.4	852	1388.4
PEI	1246.2	1851.6	605.4	1548	628.8	1770

Number of mounts (MN), intromissions (IN), and ejaculations (EN). Latency of mount (ML), intromission (IL), and ejaculation (EL). PEI: postejaculatory interval.

## Data Availability

Data is available on request.
